# New Remedies

**Published:** 1862-09

**Authors:** Samuel R. Percy


*New Remeaies, by* Samuel R. Percy, M. D., *re-printed from the American Medical Times.*

RESINA PODOPHYLLI (PODOPHYLLIN.)

*Physiological Effects.*—I am not aware that any physiological experiments that have been published have been performed on animals, either with the podophyllum root or with the resin. I have performed some few experiments on animals, but mostly to ascertain the purgative effects of the resin. With the fresh root I have tried no experiments either on man or animals, but from the descriptions found in the books, and from the relation of some few cases to me, it seems to produce great irritation of the intestinal canal, gripings, prostrating emesis, and cartharsis, an irritable and frequent pulse, and profuse salivation. These irritant effects are produced by a volatile principle existing in the green root or plant, which is mostly dissipated on drying. The effect of the green root or plant, or the fresh decoction of them, upon the mouth and salivary glands, resembles in a mild degree that of the Arum tryphyllum, and the profuse salivation produced is principally the effect of the local stimulation, for salivation is but very slightly induced by the dried root or resin, unless it is given to its emetic effect; then it acts as emetics in general, and freely increases the secretion of saliva. It has been so frequently asserted that podophyllin produces salivation that I have taken much pains to ascertain its action in this respect, and I have found, when given in pills or capsules in small and frequently repeated doses, or in one large dose, that it has no persistent sialagogue action, and no effect like mercury, producing soreness of the gums, fœtor of the breath, and profuse and continued secretion of saliva. As I before stated, when given to its nauseant or emetic effect, it always induces a free secretion of saliva, but as its emetic action passes off so does its sialagogue action also. But if the resin is given in powde r, so that it produces a local stimulation upon the glands, I have seen abundant secretio n of saliva for one or two hours. In this way it is merely a topical irritant, not a true sialagogue. There are no means of ascertaining whether the resin when passed into the stomach in capsules can be detected in the saliva, but that it exists in the saliva, when administered by the mouth in powder there can be little doubt, for the resin is soluble in the saliva.

Of the commercial podophyllin (of Messrs. Keith's manufacture) I have given two grains to a dog; in eight hours it produced three free alvine evacuations. The same dose was repeated the next day, and it acted on the bowels in three hours, and during the day caused more than a dozen evacuations. To the same dog I administered, by hypodermic injection, under the skin of the leg, one grain of podophyllin dissolved in liquor potassæ. It produced great local irritation, free purging in two hours and twenty minutes, evident colicky pains, and much tenesmus, with retching, but no vomiting.

To a man suffering with constipation of the bowels, I have sprinkled two grains of the resin in fine powder over a large indolent ulcer. It caused great pain in the ulcer, free catharsis in six hours, with nausea and severe griping pain. Within twenty-four hours it acted on the bowels seven times. The appearance of the ulcer was improved by the application.

Podophyllin, when administered to a person in health, is an efficient and certain cathartic; slow in its operation if administered in proper medicinal doses, but if administered in large doses quick and violent in its action, causing nausea, vomiting, and repeated and painful purging of mucous and billious matters. When taken in powder in moderate doses, it is not very disagreeable when first put into the mouth, but as soon as the saliva dissolves a portion of it becomes disagreeably bitter and nauseous, and the sensation it leaves in the mouth and fauces is quite unpleasant; when taken in this way, there is a free secretion of saliva for some time. When I have taken the powder finely rubbed up with sugar in this way, there is no sensation experienced in the stomach for an hour or more, excepting the first sensation of nausea from the disagreeable taste. In about an hour, if it has been taken fasting, there is an uneasy feeling in the stomach, accompanied with nausea and free salivation. This lasts for about an hour, and it feels as though a large secretion of gastric fluid was being poured out, and the stomach feels as if in a state of commotion. Soon the influence is felt in the small intestines, and unmistakable sensations of the secretion of the bile are experienced. In this stage of operation it produces on me exactly the same sensations as I experience from a full dose of calomel. The influence continues to be felt through the whole length of the intestines, producing active peristaltic motions, and the sensation as though acrid bile was freely passing. In about five hours one grain will purge me quite freely, and this is followed within two hours by two or three bilious evacuations, producing upon me the same sensations and the same bilious-appearing alvine evacuations that I experienced from the same proportionate dose of calomel. In this dose it does not gripe nor produce much tenesmus, but during the whole time of its passage through the intestines there is an unmistakable sensation of a dose of medicine producing a chologogue effect within. If the same dose (one grain) is taken immediately after eating, and protected in any way so that it does not touch the mouth, no effects whatever are felt from it for two or three hours; then the effects in the intestines above described are experienced in a very modified degree, and the result will be one copious pultaceous evacuation. The after effects in both instances are an increase of appetite, and a feeling of better health. Most persons will require a rather larger dose of the commercial article than this, and many can take three grains.

*Therapeutical Effects.*—Podophylin was first and most largely used by the "Eclectics," and many of them have written intelligently upon its therapeutic applications. By the Eclectics it has been called Vegetable Mercury, and its use has been recommended in all diseases in which mercury has been found to be of service. To a certain extent, and in some of its effects, it certainly does much resemble that drug.

Its greatest use is in that class of diseases usually called bilious disorders; that is, in those disorders where the whole digestive organs are deranged. In these disorders a dose of one, two, or three grains of the commercial podophylin will be found to excite the secretion of all the abdominal organs, acting as an efficient purgative by this increased secretion. The largest number of patients whom we are called upon to treat are suffering more or less from these disorders, and it has undoubtedly been too much the case to give some mercurial for their relief. In these disorders podophylin, combined in the manner we shall hereafter describe, is fully as efficient to cause a free secretion from the intestinal mucus membrane, and from the liver and pancreas, as any of the preparations of mercury, and it is infinitely safer. There is a very grave accusation made against our Military Army Surgeons for using too much blue pill and calomel in these disorders, and although the accusation is an unjust one against the majority of the surgeons, there are undoubtedly some against whom it is true. Our soldiers, who are so much exposed, should not use mercurials if it can be possibly avoided, and this article will, if properly given in these disorders, have a more beneficial effect, and will produce none of the evil consequences of mercury. There is but one drawback to its use, that is the inability of the patient being upon duty for ten or twenty hours after taking it, owing to the nausea and tormina it produces if given in a full dose. In some of the forms of hepatitis it is of great value, and causes a full secretion of bile, but as this is not the only indication in the acute form of the disease, it cannot be relied upon to check the inflammation. In chronic hepatitis I have found it of very great service, acting better than any other remedy I am acquainted with, as it relieves the portal circulation by its action on the secernents. In this disorder it is not necessary, in fact it is frequently injurious, to give it in large and powerfully cathartic doses. I have found it better to give it in small and frequently repeated doses upon an empty stomach, sometimes combined with veratrum or hydrocyanic acid, at other times with strychnia or capsicum. The amount of bile and intestinal mucus secretion carried off by treatment of this description is sometimes enormous.

There are few diseases in which it is of more service than habitual constipation. In this disease small doses taken with the meals (frequently in combination with strychnia and capsicum) will in the majority of instances relieve the disorder within two weeks. It needs but proper graduation to give it in just the proper proportion.

From its thorough action upon the whole intestinal mucus surface, and upon the large glands, it is one of the best eliminants in infantile convulsions. For the same reason it advantageously follows the use of anthelmintics.

But as from the experiments I have made with it I will endeavor to give its mode of action, I would rather leave its application to your own judgment. If you know its physiological and therapeutic action, you can apply it intelligently in the treatment of diseases.

